# Prevalence of factors associated with poor outcomes of hospitalized myasthenia gravis patients in Thailand

**Published:** 2014-10

**Authors:** Somsak Tiamkao, Sineenard Pranboon, Kaewjai Thepsuthammarat, Kittisak Sawanyawisuth

**Affiliations:** *From the Department of Medicine (Tiamkao, Sawanyawisuth), the Clinical Epidemiology Unit (Thepsuthammarat), Faculty of Medicine, the Nursing Division (Pranboon), Srinagarind Hospital, the Integrated Epilepsy Research Group (Tiamkao, Pranboon), the North-Eastern Stroke Research Group (Tiamkao), and the Research Center in Back, Neck Other Joint Pain and Human Performance (Sawanyawisuth), Khon Kaen University, Khon Kaen, Thailand*

## Abstract

**Objective:**

To examine the prevalence of hospitalized myasthenia gravis (MG), and to determine the factors associated with poor outcomes of hospitalized MG patients at a national level.

**Methods:**

This study was based on a retrospective design. We collected data of hospitalized MG adults recorded by the National Health Security Office, Bangkok, Thailand between October 2009 and September 2010. Clinical data and treatment outcomes were examined.

**Results:**

The total number of hospitalized MG patients was 936 cases. The prevalence rate of hospitalized MG patients was 2.17/100,000 population. The average age (SD) was 44.93 (14.16) years. Regarding the discharge status of MG patients, 845 cases (90.3%) had improved. The total hospital charge of MG patients was 64,332,806 baht (USD 2,144,426.87) or an average of 68,731.63 baht/admission (USD 2,291.05), with an average length of stay of 10.45 days. There were 3 significant factors associated with poor outcomes in hospitalized MG patients; namely, hospital category, pneumonia, and respiratory failure.

**Conclusions:**

The prevalence of admission in MG patients was 2.17 persons/100,000 population. Hospital category, pneumonia, and respiratory failure were significant factors associated with poor outcomes.

Myasthenia gravis (MG) is a neurological disease involving neuromuscular junctions. Antibodies against acetylcholine receptors in the post-synaptic membrane lead to muscle weakness. The prevalence of MG is 2-7/10,000 in the UK,^1^ and 14.2/100,000 population in the United States.^2^ Myasthenia gravis patients may need hospitalization if they have myasthenic crisis resulting in respiratory failure. Intubation and mechanical ventilation may be needed. Other treatment options include treatment by plasmapheresis or intravenous immunoglobulin (IVIG).^3^ Hospitalization of MG patients is the major cause of morbidity, and may result in high economic burdens. Requiring ventilator support and management in the intensive care unit are the main requirements in hospitalized MG patients.^4^ In Thailand, there is a lack of studies on the prevalence and also the factors associated with treatment outcomes of hospitalized MG patients at a national level. These results may be of benefit in Asian countries concerning treatment protocols and financial plans for hospitalization of MG patients. We aimed to examine the prevalence of hospitalized myasthenia gravis (MG), and to determine the factors associated with poor outcomes of hospitalized MG patients at a national level.

## Methods

This study was conducted on an adult population aged 18 years and over who were admitted to hospitals in Thailand. Data were retrieved from the national reimbursement health insurance system. The system is comprised of 3 levels of health insurance; universal coverage, social welfare, and government welfare. Universal coverage is basic health insurance for the general population, while social welfare, and government welfare are in place for people who work for private companies and government organizations. The MG diagnosis was determined using the International Classification of Diseases-10 (ICD 10) code.

Data of all admitted MG patients between October 2009 and September 2010 were collected. The data included baseline characteristics of the patients, types of hospital, hospital regions, types of insurance, complications of MG, treatments, treatment outcomes, and length of hospital stay. Thailand is divided into 4 regions geographically; northern, northeastern, central, and southern. Four categories of hospital occur in Thailand; primary, secondary, tertiary, and private. The categories of hospital are defined as follows: Primary hospital: a district hospital with a capacity of 10-30 patient beds, a sub-district health promotion hospital, or a community health center. Secondary hospital: a provincial or district hospital capable of providing services at a secondary level with 30-500 beds. Tertiary hospital: a provincial, regional, or central hospital capable of providing services at a tertiary level with over 500 beds. Private hospital: a privately owned health center. Outcomes of the study included hospital charge, length of stay, and discharge status. Discharge status as defined by the summary note of a physician was classified as improved, not improved, or dead. The latter 2 discharge states were considered poor outcomes. The study protocol was approved by the ethics committee in human research, Khon Kaen University and followed the Helsinki Declaration.

### Data analysis

The prevalence rate of hospitalized MG was calculated based on a population from Thailand who were 18 years old or over in the 2010 fiscal year. The association of length of hospital stay, and MG complications and treatment were analyzed. Factors associated with discharge status were also analyzed by descriptive statistics. Wilcoxon rank-sum and Fisher’s exact tests were applied to compare the differences in numbers and proportions between the 2 groups. Patients were divided into 2 groups by the discharge status; improved versus poor outcomes (not improved, or dead). Univariate logistic regression analyses were applied to calculate the crude odds ratios of individual variables for good discharge status. All clinically significant variables or *p*-value less than 0.20 by univariate analyses were included in subsequent multivariate logistic regression analyses. Analytical results were presented as crude odds ratios (OR), adjusted OR, and 95% confidence intervals (CI). All data analyses were performed with STATA software (StataCorp LP, College Station, Texas, USA).

## Results

The survey was conducted in the 2010 fiscal year on a population of 57,051,454 persons. Adults over 18 years of age accounted for 43,020,670 (75.4%). The total number of MG patients that were admitted was 936 or 2.17 persons/100,000 population with the highest rate found in the central region of Thailand (**Table 1**). The female:male ratio was 2.52:1 and the average age (SD) was 44.93 (14.16) years. There were 20 patients who died during admission (2.1%). Six hundred and thirty-nine patients (68.3%) had universal health coverage insurance. Five hundred and twenty-one patients (55.7%) were admitted to a tertiary care hospital. Three hundred and ninety-one patients (41.8%) lived in the central region, and a total of 845 (90.3%) patients had improved status at discharge as shown in **Table 2**. Five significant factors were found to be associated with the discharge status of the patients; insurance group, hospital category, hospital region, pneumonia, and respiratory failure (**Table 2**). There were 234 patients (25%) who required invasive mechanical ventilation, while 90 (9.6%) patients had pneumonia. Four out of 234 patients (1.7%) had respiratory failure and received IVIG therapy (**Table 3**). During admission, 145 patients (15.5%) received thymectomy, and 40 patients (4.3%) were sent for CT of the chest. The total hospital charge for all patients was 64,332,806 baht (USD 2,144,426.87) or an average of 68,731.63 baht/admission (USD 2,291.05). The average (SD) length of stay (LOS) was 10.45 (18.29) days. The LOS in patients with either pneumonia or respiratory failure was significantly longer than those who did not have these complications. The average LOS in the pneumonia group was 28.74 days, while in the none pneumonia group it was 8.50 days. The respiratory failure group had an average LOS of 22.74 days, while it was 6.35 days for the group with no respiratory failure. A similar LOS was found for the treatment with IVIG group compared with the group who did not receive IVIG (48 versus 22.3 days). Of the 4 patients who received IVIG, one patient had pneumonia and was treated with IVIG, but this did not correlate with pneumonia complications (*p*=0.728). There were 3 significant factors associated with poor outcomes in hospitalized MG patients by multivariate logistic analysis; namely, hospital category, pneumonia, and respiratory failure (**Table 4**). The latter 2 factors were positively associated with poor outcomes with adjusted odds ratio of 2.20 and 4.67.

**Table 1:** Prevalence rate of hospitalized myasthenia gravis patients classified by regions of Thailand.

Regions	Population ages 18 and over	Number of admissions by primary diagnosis (G70)	
		Number	Patient/100,000
Northern	8,871,705	220	2.48
Northeast	15,964,936	276	1.73
Central	11,896,555	391	3.39
Southern	6,287,474	49	0.78
Total	43,020,670	936	2.17

G70 - ICD10 code for myasthenia gravis

**Table 2:** Factors affecting discharge status of hospitalized myasthenia gravis patients (N=936).

Variable	Discharge Status				*P*-value
	Improved	Not improved	Dead	Total	
	n (%)				
***Gender***					0.399
Male	242 (91.0)	21 (7.9)	3 (1.1)	266 (28.4)	
Female	603 (90.0)	50 (7.5)	17 (2.5)	670 (71.6)	
***Age***					0.072
18-29	138 (91.4)	12 (7.9)	1 (0.7)	151 (16.1)	
30-39	201 (91.8)	16 (7.3)	2 (0.9)	219 (23.4)	
40-49	204 (89.1)	22 (9.6)	3 (1.3)	229 (24.5)	
50-59	177 (90.3)	14 (7.1)	5 (2.6)	196 (20.9)	
60-69	93 (89.4)	5 (4.8)	6 (5.8)	104 (11.1)	
70-79	23 (88.5)	1 (3.8)	2 (7.7)	26 (2.8)	
80+	9 (81.8)	1 (9.1)	1 (9.1)	11 (1.2)	
***Insurance groups***					0.040
Government welfare	95 (90.5)	6 (5.7)	4 (3.8)	105 (11.2)	
Social welfare	183 (95.3)	7 (3.6)	2 (1.0)	192 (20.5)	
Universal coverage	567 (88.7)	58 (9.1)	14 (2.2)	639 (68.3)	
***Hospital category***					<0.001
Primary	72 (72.0)	27 (27.0)	1 (1.0)	100 (11.0)	
Secondary	175 (87.1)	22 (10.9)	4 (2.0)	201 (21.0)	
Tertiary	491 (94.2)	18 (3.5)	12 (2.3)	521 (56.0)	
Private	107 (93.9)	4 (3.5)	3 (2.6)	114 (12.0)	
***Regions***					<0.001
Northern	197 (89.5)	19 (8.6)	4 (1.8)	220 (23.5)	
Northeast	240 (87.0)	34 (12.3)	2 (0.7)	276 (29.5)	
Central	367 (93.9)	11 (2.8)	13 (3.3)	391 (41.8)	
Southern	41 (83.7)	7 (14.3)	1 (2.0)	49 (5.2)	
***Pneumonia***					<0.001
No	778 (92.0)	56 (6.6)	12 (1.4)	846 (90.4)	
Yes	67 (74.4)	15 (16.7)	8 (8.9)	90 (9.6)	
***Respiratory failure***					<0.001
No	660 (94.0)	41 (5.8)	1 (0.1)	702 (75.0)	
Yes	185 (79.1)	30 (12.8)	19 (8.1)	234 (25.0)	
***Intravenous immunoglobins***					0.999
No	840 (90.2)	71 (7.6)	20 (2.1)	931 (99.5)	
Yes	5 (100.0)	0 (0.0)	0 (0.0)	5 (0.5)	

**Table 3:** Length of stay of hospitalized myasthenia gravis patients by various factors.

Factors	Count	Length of stay		*P*-value
		Mean	SD	
***Pneumonia***				<0.001
No	846	8.50	14.31
Yes	90	28.74	34.59
***Respiratory failure***				<0.001
No	702	6.35	10.22
Yes	234	22.74	28.74
***IVIG treatment***	234	22.74	28.74	0.142
No	230	22.30	28.35
Yes	4	48.00	44.12

IVIG - intravenous immunoglobulin

**Table 4:** Significant factors associated with poor outcome at discharge of hospitalized myasthenia gravis patients by multivariate logistic analysis.

Variable	Adjusted odds ratio	95% confidence interval	*P*-value
***Hospital category***			<0.001
Primary	1.00		
Secondary	0.24	0.12-0.46	
Tertiary	0.09	0.04-0.18	
Private	0.28	0.09-0.84	
***Pneumonia***			0.027
No	1.00		
Yes	2.20	1.10-4.39	
***Respiratory failure***			<0.001
No	1.00		<0.001
Yes	4.67	2.66-8.21	

Adjusted for gender, age, insurance groups, hospital levels, regions, having pneumonia, having respiratory failure, and intravenous immunoglobulin treatment

## Discussion

There is a lack of studies on the prevalence of hospitalized MG in Thailand. In our study, the admission rate was 2.17 patients/100,000 population. In the United States, the incidence rate of MG inpatients is higher among black ethnic females (0.01/1,000 person/year) than white females (0.009/1,000 person/year).^5^ The prevalence rate of MG in Taiwan is 14/100,000, and both Estonia, and Australia have recorded rates of 11.7/100,000.^6^-^8^ The prevalence rates shown in previous studies were not the prevalence of admission as was examined in the present study.

We found that the mortality rate was 2.1%, which is similar to reports from the US.^5^ The mortality rate in hospitals in the US is 2.2%, and increased to 4.4% in MG with crisis. The survival rates of MG after diagnosis at year 3 were 85%, at year 5 were 81%, at year 10 were 69%, and at year 20 were 63% in Denmark. One systematic review on a population study showed that the mortality rate was 0.1-0.9/millions person/years.^9^ Generally, age was shown to be a prognostic factor specifically in patients equal to or greater than 60 years old. In the present study, the age group of more than 60 years showed a dramatically higher mortality rate (**Table 2**). The mortality rates of the sixth decade were 5.8%, of the seventh decade were 7.7%, and of the eighth or more decades were 9.1%. However, the mortality rate from MG was quite similar when compared with stroke, which had a mortality rate of 9.9%.^10^

The vast majority of admitted MG patients were female (71.6% verus 28.4%), which corresponds to disease prevalence.^5^ Two factors that significantly prolonged hospital stay were having pneumonia or respiratory failure (**Table 2**). With respect to these 2 factors, having respiratory failure was the major reason for hospitalization in MG patients (234 versus 90 patients). Of the patients who had respiratory failure, only 4 patients received IVIG treatment. The durations of hospital stay in patients who received IVIG were not statistically different from patients who did not receive treatment (**Table 3**). Having pneumonia or respiratory failure prolonged hospital stay approximately 3-4 times compared with patients who did not have these conditions. Patients who receive IVIG, even though they show a good response, pay a very high cost, which ranges from 180,000 to 250,000 baht (USD 6,000- 8,333).^2^ In the United States, hospitalized MG patients spent USD 20,190 per admission, and the average cost was USD 109,463 ± 57,303 if one received IVIG.^11^ In Taiwan, an MG outpatient pays 1,894 New Taiwan Dollars (NTD, 1 USD = 30-34 NTD), while inpatients’ expense amounts to an average total of 107,976 NTD /patient or 42,079 NTD/patient/year.^6^

Having pneumonia resulted in a somewhat higher mean duration of hospital stay than having respiratory failure (28.74 versus 22.74 days). Both factors were also significantly associated with discharge status (**Table 4**). Unlike previous studies, we did not find that age and gender were significant prognostic factors in relation to discharge status. Age over 60 years and male gender were found to be poor prognostic factors.^12^ These findings may be due to analysis of a different study population. Previous studies included all MG patients, while the present study enrolled admitted MG patients at a national level. We also found that hospital levels and regions were significantly related with discharge status (**Table 2**). Death and no improved status were found more in primary hospitals where medications and facilities are limited. Physicians in tertiary care hospitals may have gained more knowledge in management because they are neurologists. Patients with universal health insurance coverage had limited use of medications resulting in poorer outcomes. Insurance types, and hospital regions were statistically different factors among various outcomes. However, only hospital category, having pneumonia, and having respiratory failure were significantly independent factors associated with poor outcomes (**Table 4**). Having pneumonia and respiratory failure increased the risk of poor outcomes by 2.2 and 4.67 times.^13^

The main limitation of this study is that data were acquired from summary charts of admitted patients for reimbursement. Therefore, specific details of each patient such as disease severity or patient compliance were not available for analysis. All data were collected from the reimbursement database system for inpatients only; there were no data for outpatient MG treatment. Results from this study can provide a sound foundation for the planning of appropriate treatment of MG patients in the future in Thailand and other Asian countries.

In conclusion, the prevalence of admission in MG patients was 2.17 persons/100,000 population. Hospital category, having pneumonia, and having respiratory failure were significant factors associated with poor outcomes. Further prospective clinical studies are needed to confirm the results of this study.
